# Nonalcoholic fatty pancreatic disease and cardio-metabolic risk: is there is a place for obstructive sleep apnea?

**DOI:** 10.1186/1475-2840-13-29

**Published:** 2014-01-30

**Authors:** Aibek E Mirrakhimov

**Affiliations:** 1 Kyrgyz State Medical Academy named by I.K. Akhunbaev, Akhunbaev street 92, Bishkek 720020, Kyrgyzstan

**Keywords:** Obstructive sleep apnea, Non-alcoholic fatty liver disease, Non-alcoholic fatty pancreatic disease, Cardiometabolic risk, Pancreatitis, Pancreatic cancer

## Abstract

**Background:**

Obstructive sleep apnea is a common disorder acting as a risk factor for the development and progression of cardiometabolic derangements including non-alcoholic fatty liver disease. Recent research data suggest that non-alcoholic fatty pancreatic disease may be a more sensitive marker than non-alcoholic fatty liver disease for early subclinical metabolic risk and may contribute to the progression of subclinical disease to overt type 2 diabetes mellitus.

**Presentation of the hypothesis:**

We postulate that obstructive sleep apnea may be a risk factor for non-alcoholic fatty pancreatic disease. It is well known that intermittent hypoxia related to obstructive sleep apnea leads to hormonal derangements. Excessive lipolysis, enhanced lipid synthesis and systemic and local inflammation may favor ectopic fat deposition similarly to non-alcoholic fatty liver disease. Furthermore, it is possible that obstructive sleep apnea can lead to pancreatic beta cell damage via intermittent hypoxia.

**Testing of the hypothesis:**

Future research should focus on the following: first, whether non-alcoholic fatty pancreatic disease is an independent risk factor for the development of metabolic disease including diabetes mellitus or is a simple consequence of obesity; second, the prevalence of non-alcoholic fatty pancreatic disease among people with obstructive sleep apnea and vice versa, which should be compared to the prevalence of these diseases in general population; third, whether coexistence of these conditions is related to greater cardiometabolic risk than either disease alone; and fourth, whether the treatment of obstructive sleep apnea will translate into the resolution of non-alcoholic fatty pancreatic disease.

**Implications of the hypothesis:**

If proven, this hypothesis will provide new knowledge on the complex interplay between various metabolic insults. Second, screening for NAFPD may identify individuals at risk for developing type 2 diabetes mellitus for targeted prevention. Third, screening for the presence of non-alcoholic fatty pancreatic disease in patients with obstructive sleep apnea may help to decrease the incidence of diabetes mellitus through a targeted prevention.

## Introduction

Obstructive sleep apnea (OSA) is a disorder that is characterized by complete or partial breathing disturbances during sleep with a minimum prerequisite frequency of five events per hour, each of which should last for at least 10 seconds [1]. These events occur as a result of anatomical and/or physiological features of the upper airways and their neural control or due to craniofacial parameters [2]. Its prevalence varies in epidemiological surveys, which can be attributed to the different populations being studied, as well as to whether or not daytime sleepiness is considered an inclusion criterion for OSA diagnosis [3]; therefore, the percentage of the affected population may be much larger than reported.

Recent data suggests that OSA is strongly associated with cardiovascular, metabolic and even malignant disease and may be an independent risk factor for their occurrence [4-11]. Nonalcoholic fatty liver disease (NAFLD) is considered to be a manifestation of metabolic syndrome and is strongly related to excessive cardiovascular risk as well as renal disease [12-14]. On the other hand, NAFLD may independently contribute to insulin resistance [15] and vascular stiffness [16]. Furthermore, NAFLD is considered to be among the leading causes of liver cirrhosis and hepatocellular carcinoma [17]. Robust scientific data indicates that OSA is strongly related to NAFLD independently from traditional risk factors [5,18-20].

More recently, however, the fatty infiltration of pancreas or nonalcoholic fatty pancreatic disease (NAFPD) was shown to correlate with metabolic risk [21]. Indeed, NAFPD was shown to precede the development of NAFLD, and, thus, may serve as a metabolic risk marker [22].

From a theoretical standpoint, the presence of NAFPD may explain why obese patients have worse outcomes than their lean counterparts in acute pancreatitis [23]. It is possible that NAFPD may follow the route of NAFLD in terms of association with the development of organ-specific cancer. However, a single-center study performed by Sipe et al. did not find an association between NAFPD and pancreatic cancer [24]. At the same time, obesity and a fat-rich diet were shown to be strongly related to an increased risk of pancreatic cancer [25-27]. Indeed the above interrelationship between an unhealthy metabolic profile and lifestyle may be mediated via NAFPD. Whether, on a biological level NAFPD behavior vary from simple steatosis to a more aggressive steatopancreatitis similarly to NAFLD spectrum is a matter for future research. If so, steatopancreatitis may increase the risk of pancreatic cancer and explain the association between obesity and pancreatic cancer [25-27].

We hypothesize that OSA is an extra hit for the development of NAFPD and subsequent metabolic risk. It is interesting to note that OSA may mediate hypoxic damage to pancreatic beta-cells and, thus, lead to type 2 DM [28]. Below we will formulate our hypothesis. Second, we will review our current understanding of how OSA leads to ectopic fat deposition, which is mainly based on our understanding of NAFLD. Third, the potential metabolic implications of NAFPD will be discussed. Finally, we will discuss what steps need to be taken by the research community to investigate whether OSA is a mediator of cardiovascular and metabolic risk via its relationship with NAFPD.

## Hypothesis

We hypothesize that OSA may mediate its detrimental cardiovascular and metabolic effects through the development of ectopic fat deposition in pancreatic tissue.

### OSA and ectopic fat deposition: a model of NAFLD

Recent data strongly suggest that OSA is an important contributing factor for the occurrence of dyslipidemia [29,30]. The pathogenesis of the above association is mediated by at least three major processes such as enhanced lipolysis in adipose tissue, decreased lipid clearance and upregulated lipid synthesis in the liver. A review of the literature on NAFLD and OSA by Mirrakhimov and Polotsky noted factors that play key pathobiological roles in the pathogenesis of NAFLD in the setting of OSA [5]. Intermittent hypoxia (IH) is a key mediator of OSA and leads to various alterations in biological homeostasis [31]. First of all, it activates the sympathetic nervous system via the carotid body chemoreceptors, which in turn leads to fat degradation (lipolysis) from adipose tissue. Free fatty acids (FFA) compose the triglycerides (a major form of fat storage in the body; another major constituent of triglycerides is glycerol), which are transported to different organs to serve as fuel. However, FFA can lead to steatosis and organ inflammation, such as in the setting of NAFLD. At the same time, IH activates hypoxia inducible factors 1 and 2 (HIF-1 and HIF-2 respectively), which lead to decreased lipid metabolism in the liver, increased hepatic fat synthesis, upregulated liver inflammation and fibrosis. The HIF family is thought to play a major role both in physiologic adaptation as well as in the pathophysiology of disease states [32]. Indeed, the activation of HIF seems to play a key role in the development and pathogenesis of cancer [32-35]. Therefore, the previously described relationship between OSA and cancer [8-11] may be mediated via the HIF pathway.

More recently a study by Minville et al. showed that OSA and more specifically, nocturnal hypoxia was demonstrated to be independently associated with liver steatosis in obese individiuals further supporting the independent interrelationship between OSA and metabolic diseases [19]. Furthermore, a recent meta-analysis by Sookoian and Pirola showed that patients with OSA have greater transaminase values, higher prevalence of NAFLD and liver fibrosis compared to individuals without OSA [20].

An important question is whether OSA is independently (adjusted for confounders such as body mass index (BMI), smoking, age, gender, alcohol intake, family history etc.) associated with organ-specific cancers [11]. Of particular importance is to study whether OSA is linked to hepatocellular carcinoma via its association with the occurrence of NAFLD.

We hypothesize that OSA may contribute to the occurrence of NAFPD in a similar fashion as it does to NAFLD. Future studies should assess whether OSA is a true independent risk factor for the occurrence of NAFPD, given that NAFPD may precede NAFLD [20] and thus, may be an early marker of excess in cardiac and metabolic risk. Therefore, identifying patients with NAFPD and OSA even without clinically overt metabolic disease such as metabolic syndrome or type 2 DM would necessitate aggressive risk reduction in order to prevent the future development of type 2 diabetes mellitus, which is a major contributor to excessive morbidity and mortality worldwide.

### NAFPD and metabolic risk

The relationship between metabolic health and fatty infiltration of the pancreas is a well-known phenomenon, which was first described in 1926 by Schaefer, who showed a correlation between the weight of the human body and that of the pancreas [36]. In 1933 Ogilvie demonstrated that obese cadavers had heavier pancreatic glands than non-obese cadavers [37]. Both of these pioneer studies suggested that pancreatic weight goes in parallel with body weight. It seems that this association may now be explained by ectopic fat deposition in pancreatic tissue.

Several animal studies have investigated the effects of fat deposition in pancreas. Shimabukuro et al. demonstrated that FFA induced apoptosis of pancreatic beta- cells (endocrine cells producing insulin) in Zucker diabetic fatty rats [38]. Mathur et al. showed that obese mice had heavier pancreata with excessive fat infiltration and enhanced production of pro inflammatory cytokines [39]. Lee et al. demonstrated fat accumulation in both the exocrine and endocrine pancreas in a rat model [40]. These researchers speculated that fatty infiltration of the endocrine pancreas may impair insulin secretion and thus lead to type 2 DM.

Tushuizen et al. studied 12 males with type 2 DM and 24 age- and BMI-matched non-diabetic men [41]. These researchers demonstrated that pancreatic fat was independently (adjusted for BMI, fasting plasma glucose and triglycerides) associated with worse pancreatic beta cell function. Lee et al. showed that NAFPD was associated with higher levels of insulin resistance, visceral adiposity, triglycerides and alanine aminotransferase compared to normal pancreata [22].

Subsequently, scientific studies showed that increased age, obesity, visceral steatosis, hypertension and type 2 DM were associated with the presence of NAFPD [42-45]. Nevertheless, it is essential to admit that NAFPD is understudied entitiy and not much is known about the natural course of NAFPD. Available evidence suggests that men have a higher prevalence of NAFPD compared to women with the same BMI, insulin resistance and fat intake [46,47]. Certain groups may also have a greater prevalence of NAFPD. For example it was shown that Hispanics had greater pancreatic fat content than African-Americans [48]. These observations may explain why men and certain ethnic groups have increased metabolic risk profiles.

Most studies to date have investigated patients at risk for type 2 DM and, thus, the prevalence may be overestimated. Several imaging modalities can be of use in quantifying pancreatic fat such as trans abdominal and trans esophageal ultrasound, computed tomography and magnetic resonance tomography [44-46,49-51]. However, NAFPD is currently the focus of research rather than the clinical community given scarce data on natural history and information on whether screening for NAFPD will translate into decreased morbidity and mortality.

In a pig model Hanukkainen et al. demonstrated that pancreatic fat is associated with insulin resistance and liver fat content [52]. However, van Geenen et al. showed that the association between pancreatic fat and liver fat is mediated via central obesity [53]. Heni et al. demonstrated that pancreatic fat was negatively associated with insulin secretion in patients with pre-diabetes [54]. These researchers suggested that this effect may contribute to the progression of pre-diabetes to overt type 2 DM. van der Zijl et al. also demonstrated that patients with pre-diabetes had greater pancreatic fat content [55]. However, this group was unable to demonstrate a relationship between pancreatic fat and insulin secretion. On the other hand, insulin resistance was found to be strongly associated to the presence of pancreatic fat [56]. Sepe et al. found a close interrelationship between NAFPD and metabolic syndrome [24]. However, no correlation was found between NAFPD and pancreatitis, pancreatic cancer and DM.

More recently two studies from Taiwan were published on the association between NAFPD and metabolic risk. In a first study, Wu and Wang confirmed an intricate association between NAFPD and metabolic risk factors [21]. They retrospectively studied 557 subjects without known hypertension and DM for the prevalence of NAFPD. 12.9 % of subjects were found to have NAFPD via transabdominal ultrasonography. Older age, greater BMI, abdominal waste circumference and metabolic variables such as glycated hemoglobin, lipid parameters and systolic blood pressure were associated with the presence of NAFPD. In a second study, Ou et al. enrolled 7,464 individuals to assess prevalence of NAFLD and NAFPD [57]. These researchers found that NAFPD was associated with pre-diabetes and DM after adjustment for cardiometabolic variables. It is interesting to note that NAFPD was only found to be associated with the presence of pre-diabetes and DM in male subjects, but not in females. The lack of association between female gender, NAFPD and metabolic risk may be explained by the study methodology, lower prevalence of NAFPD compared to NAFLD and possible gender specific patterns of ectopic fat deposition. A summary of key clinical studies investigating fatty pancreas is presented in Table 1.

**Table 1:** Summary of human studies investigating pancreatic fat deposition

**First author and year**	**Country**	**Study design**	**Population studied**	**Findings**
Al-Haddad et al. 2009 [44].	USA	Case control study using endoscopic ultrasound.	120 subjects (60 females). 60 subjects were found to have evidence of fatty pancreas.	Patients with fatty pancreas had greater BMI (mean BMI 31.7 kg/m^2^ vs. BMI 25.4 of people without fatty pancreas), higher prevalence of fatty liver (57% vs 5%) and excessive alcohol use.
Lee et al. 2009 [22].	South Korea	Case control study using trans abdominal ultrasound and computed tomography.	293 subjects from obesity clinic (166 females). Patients with diabetes mellitus, thyroid disease, liver disease, pancreatic disease, renal disease and individuals with excessive alcohol use were excluded from the study.	180 subjects were found to have NAFPD. Male gender, greater BMI, waist circumference, visceral fat, alanine aminotransferase levels, triglyceride levels, fasting insulin levels and insulin resistance were strongly associated with NAFPD.
Choi et al. 2010 [45].	South Korea	Case control study using endoscopic ultrasound.	284 individuals (182 females). Subjects with history of pancreatic disease, hepatobiliary disease or gastrointestinal malignancy were excluded. 110 patients were found to have evidence of fatty pancreas.	Presence of fatty liver, visceral fat, hypertension, male gender and older age were associated with ultrasonographic evidence of fatty pancreas.
Heni et al. 2010 [54].	Germany	Cross sectional study using magnetic resonance imaging and magnetic resonance spectroscopy.	51 individuals (26 females) at risk for type 2 DM. 23 patients (14 females) were found to have pre-diabetes.	Greater BMI, waist circumference and visceral adipose tissue were associated with higher pancreatic fat content. Pancreatic fat was negatively associated with decreased insulin secretion in patients with pre-diabetes.
Sepe et al. 2011 [24].	USA	Prospective case control study using endoscopic ultrasound.	230 subjects (114 females) underwent endoscopic ultrasound. 64 individuals were found to have fatty pancreast (37 females).	Increased BMI and fatty liver were strongly associated with ultrasonographic evidence of fatty pancreas. No association was found between pancreatitis, pancreatic cancer and fatty pancreas.
Rossi et al. 2011 [47].	Italy	Cross sectional study using magnetic resonance imaging.	41 individuals (21 females). Subjects with fasting plasma glucose ≥7.0 mmol/l (≥126 mg/dl) and drinking >30 g/day for men and >20 g/day for women were excluded. 38 subjects were found to be obese (20 females).	Obese individuals had greater pancreatic fat content as well as other metabolic derangements such as visceral adiposity and fatty liver. Obese women had statistically lower amount of pancreatic and liver fat, visceral adiposity despite similar BMI. Visceral adipose tissue was found to be the strongest predictor for pancreatic and liver fat deposition.
Wu and Wang 2013 [21].	Taiwan	Cross-sectional study using trans abdominal ultrasound.	557 individuals (342 females) without history of hypertension and DM. 72 subjects (42 females) were found to have fatty pancreatic disease.	Older age, higher BMI and waist circumference, glycemic control, lipid parameters and systolic blood pressure were associated with the presence of fatty pancreas.
Ou et al. 2013 [57].	Taiwan	Cross-sectional study using trans abdominal ultrasonography.	7,464 subjects (2871 females). 483 patients were found to have DM (155 females). 18.1% of men and 14.2% of women were found to have fatty pancreas.	Fatty pancreas was found to be independently associated with pre-diabetes and DM in males.

*Abbreviations*: *BMI* body mass index, *DM* diabetes mellitus, *NAFPD* non-alcoholic fatty pancreatic disease.

Future research is needed to clarify whether NAFPD is a simple consequence of obesity or indeed, an independent player in the development of metabolic complications such as type 2 DM. It will also be interesting to investigate whether the presence of NAFPD is related to the severity of clinical pancreatitis. Of greater importance is whether NAFPD represents a risk factor for the occurrence of exocrine pancreatic cancer.

### Implication of the hypothesis

We postulate that NAFPD is an early marker of increased cardiac and metabolic risk. NAFPD may precede clinically overt metabolic disease (such as NAFLD, metabolic syndrome and type 2 DM) and, thus, would be a useful marker of cardiometabolic risk in any individual. Therefore, identifying such individuals is of paramount importance since early intervention may reduce the burden of type 2 DM and cardiovascular disease. On the other hand, OSA was shown to be a risk factor for a myriad of cardiac and metabolic complications such as cardiovascular disease, hyperlipidemia, type 2 DM and NAFLD. Of particular note, NAFLD is a spectrum of metabolic syndrome and is related to a greater risk of vascular disorders. It was reported that OSA is a contributing factor for the development of NAFLD, and through this mechanism may lead to a greater risk of type 2 DM. Furthermore, OSA may exert hypoxic damage to pancreatic β-cells and lead to the occurrence of type 2 DM in susceptible individuals.

Further studies are needed to clarify whether OSA is related to NAFPD. Studies assessing the prevalence of NAFPD in patients with OSA (and vice versa) may be needed initially. The results of such studies should be adjusted for common confounders such as age, gender, BMI and obesity, metabolic syndrome, type 2 DM, hyperlipidemia and alcohol intake. Subsequently, longitudinal studies of OSA patients will be needed to assess the impact of OSA on pancreatic fatty infiltration and the natural history of NAFPD. Studying the natural history of NAFPD (with and without concomitant OSA) is of paramount importance and such research should give answers to the following question: is NAFPD truly a benign disease or is it associated with subclinical pancreatic inflammation and a resultant increase in the severity of clinical cases of acute pancreatitis, pancreatic insufficiency (both exocrine and endocrine) and pancreatic cancer. In turn, it should be determined whether OSA is a key player in the development of the above complications.

These research questions somewhat parallel those which have been asked about NAFLD, a sister disorder of NAFPD. NAFLD was found to be a non-benign disease, with some patients progressing to liver cirrhosis and hepatocellular cancer. Whether the same is true for NAFPD is a matter for future research.

## Conclusion

OSA is a common disorder acting as a risk factor for the development and progression of various cardiometabolic derangements. Of note, OSA seems to be an independent risk factor for the development of NAFLD. Recent research data suggest that NAFPD may be a more sensitive marker for early subclinical metabolic risk than NAFLD and may contribute to the progression of subclinical disease to overt type 2 DM. We postulate that OSA may be a risk factor for NAFPD as well. OSA may mediate NAFPD via tissue hypoxia, lipolysis and fat deposition in pancreatic tissue with resultant derangements in pancreatic function. Future studies should investigate the association between OSA and NAFPD as well as give us prospective data on the natural history of NAFPD.

## Abbreviations

BMI: Boby mass index; DM: Diabetes mellitus; FFA: Free fatty acids; HIF: Hypoxia inducible factor; IH: Intermittent hypoxia; NAFLD: Non-alcoholic fatty liver disease; NAFPD: Non- alcoholic fatty pancreatic disease; OSA: Obstructive sleep apnea.

## Competing interests

The authors declare that they have no competing interests.
